# Evaluation of RNA purification methods by using different blood stabilization tubes: identification of key features for epidemiological studies

**DOI:** 10.1186/s13104-020-04943-4

**Published:** 2020-02-18

**Authors:** J. A. Carrillo-Ávila, R. de la Puente, P. Catalina, J. D. Rejón, L. Espín-Vallejo, V. Valdivieso, R. Aguilar-Quesada

**Affiliations:** 1 Andalusian Public Health System Biobank, Coordinating Node, Granada, Spain; 2 Academia T de Student, Calle Conde Cifuentes, 33, 18005 Granada, Spain; 3 Parque Tecnológico Ciencias de la Salud, Centro de investigación Biomédica, Andalusian Public Health System Biobank, Avda. del Conocimiento s/n, 18016 Granada, Spain

**Keywords:** RNA quality, Biomarker, Liquid biopsy, Pre-analytics, Biobank

## Abstract

**Objective:**

Peripheral blood is the most promising source of RNA biomarkers for diagnostic and epidemiological studies, because the presence of disease and prognostic information is reflected in the gene expression pattern. Quality RNA is used by a number of different downstream applications, so the selection of the most appropriate RNA stabilization and purification method is important. We have analyzed the RNA purified from 300 blood samples from 25 donors processed by two technicians using three methodologies with Tempus and PaxGene tubes.

**Results:**

The best quality sample results were obtained with the Tempus Spin RNA Isolation Kit and the PaxGene Blood miRNA Kit, although larger amounts of RNA were obtained with the Tempus Spin RNA Isolation Kit. Lower Cq values were observed for RNA and miRNA genes in samples that were tested with PaxGene Blood miRNA Kit and Tempus Spin RNA Isolation Kit respectively. We identify the Tempus Spin RNA Isolation Kit as the most robust methodology, whilst the MagMax for Stabilized Blood Tubes RNA Isolation Kit showed the most instability. For biobanks, which process a large cohort and conduct epidemiological studies, the Tempus Spin RNA Isolation Kit is the most appropriate methodology. The study demonstrates the robustness of real-life procedures.

## Introduction

Peripheral Blood (recently named *liquid biopsy*) is routinely collected by biobanks for biomarker analysis for scientific research projects, epidemiological studies and medical applications [1–3].

The major limitation associated with RNA is the phenomenon of RNA degradation during blood collection and storage [4]. Both the sample collection and the RNA purification procedures influence the data outcome [5]. To minimize the degradation, different RNA stabilization technologies have been developed [6, 7]. Tempus and PaxGene tubes are commonly-used for blood collection. The use of a stabilizer is mandatory if one wishes to maintain the gene expression profile. Different suppliers have released kits based that can be coupled to Tempus and/or PaxGene tubes [8]. A number of previous studies have evaluated different RNA extraction methodologies [9–11], but the results are difficult to interpret because replicas of the same samples aren’t used during the comparison of methodologies. Different tubes demonstrate different levels of stabilization efficiency depending on the specific target, so the selection of the best methodology is fundamental to any reliable further analysis [12].

In this paper, we present an unpublished comparison of three blood RNA extraction methodologies. Although there exist research studies that have analyzed the main characteristics of these methodologies [10, 11], there are currently no studies that compare these methodologies together, and analyzed the methods reproducibility and robustness. The results must be taken into account for large scale studies and biobanking purposes.

## Main text

### Methods

#### The study design

Blood samples were collected from 25 healthy donors. The donors were recruited by the Andalusian Public Health System Biobank (Granada, Spain). Twelve samples from each donor were collected: four blood samples that were collected in PaxGene tubes (2.5 ml; Preanalytix, Switzerland), and eight samples in Tempus tubes (3 ml; by Life Technologies, Germany).

**Fig. 1:**
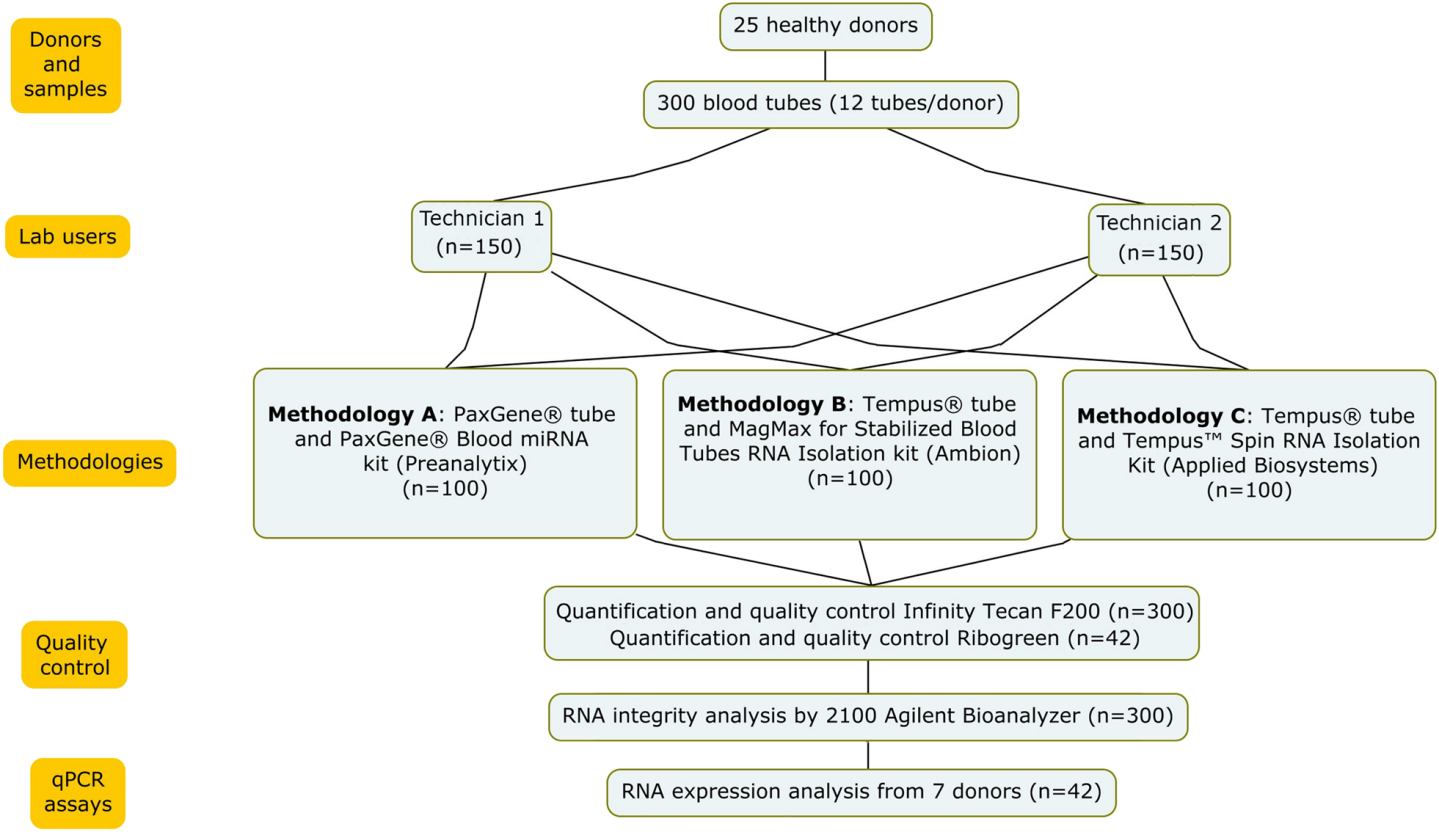
Study design: 300 samples were extracted from 25 donors. 2 sample replicas were processed from each donor by both technicians with three different methodologies. Quantification was performed for all samples by spectrofotometry (Infinity Tecan F200). A selection of the samples were also quantified by Ribogreen technique (n = 42). Expression analysis was performed for selected samples for 3 mRNAs and 3 miRNAs

The potential influence that individual technicians may have over the outcome of the sample analysis was evaluated. Consequently, two technicians processed 150 blood tubes, each using the three extraction methodologies mentioned above. Duplicate samples were processed for the 25 donors (n = 50), to check the reproducibility of each technicians’ with regards to the three tested extraction methodologies (n = 150) (Fig. 1).

#### RNA extraction

Three extraction methodologies were evaluated. For each donation, the RNA was extracted from four blood tubes by each methodology, so that duplicate samples could be processed for the 25 donors (n = 50) by each technician.

*Methodology A:* PaxGene^®^ tubes and the PaxGene^®^ Blood miRNA Kit (Preanalytix, Switzerland). The PaxGene tubes were semi-automated processed using the Qiacube robot (Qiagen) [13].

*Methodology B:* Tempus^®^ tubes and the MagMax for Stabilized Blood Tubes RNA Isolation Kit (ThermoFisher, USA) [14] and a DynaMag-2 stand (Life Technologies, USA).

*Methodology C:* Tempus^®^ tubes and the Tempus™ Spin RNA Isolation Kit (Applied Biosystems, USA) [15]. No optional DNase treatment was performed.

#### RNA quantification and quality analysis

The RNA yield and its purity were measured using an Infinite F200 spectrophotometer (Tecan, Switzerland). The concentration was measured against the A_260_ nm value, while A_260_/A_280_ nm and A_260_/A_230_ ratios were used to calculating the purity.

Forty-two samples from 7 donors were measured by fluorimetry technique using the Quant-iT™ RiboGreen™ RNA Assay Kit (ThermoFisher Scientific, EEUU) (Fig. 1).

#### RNA integrity analysis

Samples RNA integrity was measured using the Bioanalyzer 2100 (Agilent Technologies Ind., USA) and the Agilent RNA 6000 Nano Kit (Agilent Technologies Inc.). The RNA integrity (RIN value) was calculated.

#### cDNA synthesis

Samples from 7 donors (n = 42) were randomly selected for cDNA synthesis and expression analysis. Samples were extracted by the two technicians using the three methodologies. RNA was reverse-transcribed using miScript II RT Kit (Qiagen) (Additional file 1).

#### miRNA qPCR

Expression assays were generated in triplicate for three miRNAs (miR16, miR26, and miR30), with the miScript SYBR Green PCR Kit (Qiagen) (Additional file 1).

#### mRNA qPCR

Expression assays were generated in triplicate for three mRNAs (18S rRNA [16], ACTB [17], and GAPDH) [18]). The FastStart Essential DNA Green Master Kit (Roche LifeScience) was used for amplification (Additional file 1).

#### DNase treatment

An extraordinary DNase treatment was carried out on 20 µl of selected RNAs using the RNase-Free DNase Set (Qiagen).

#### Statistical analysis

A statistical analysis was performed using the IBM SPSS Statistics program 17.0.3 (SPSS, Inc., Chicago, IL). A descriptive statistic analysis was also performed with the XLSTAT v2017.6 program. The ANOVA test (p < 0.05) and the ‘post hoc test after ANOVA’—Scheffé’s method—(p < 0.05) was applied to compare the mean differences between methodologies. Grubbs test was applied for outliers identification. Box plots were constructed with the XLSTAT v2017.6 program for the visualisation of replicas variability.

### Results

Samples were extracted using three methodologies. The main characteristics are summarized in Additional file 2: Table A.

#### RNA yield

**Fig. 2:**
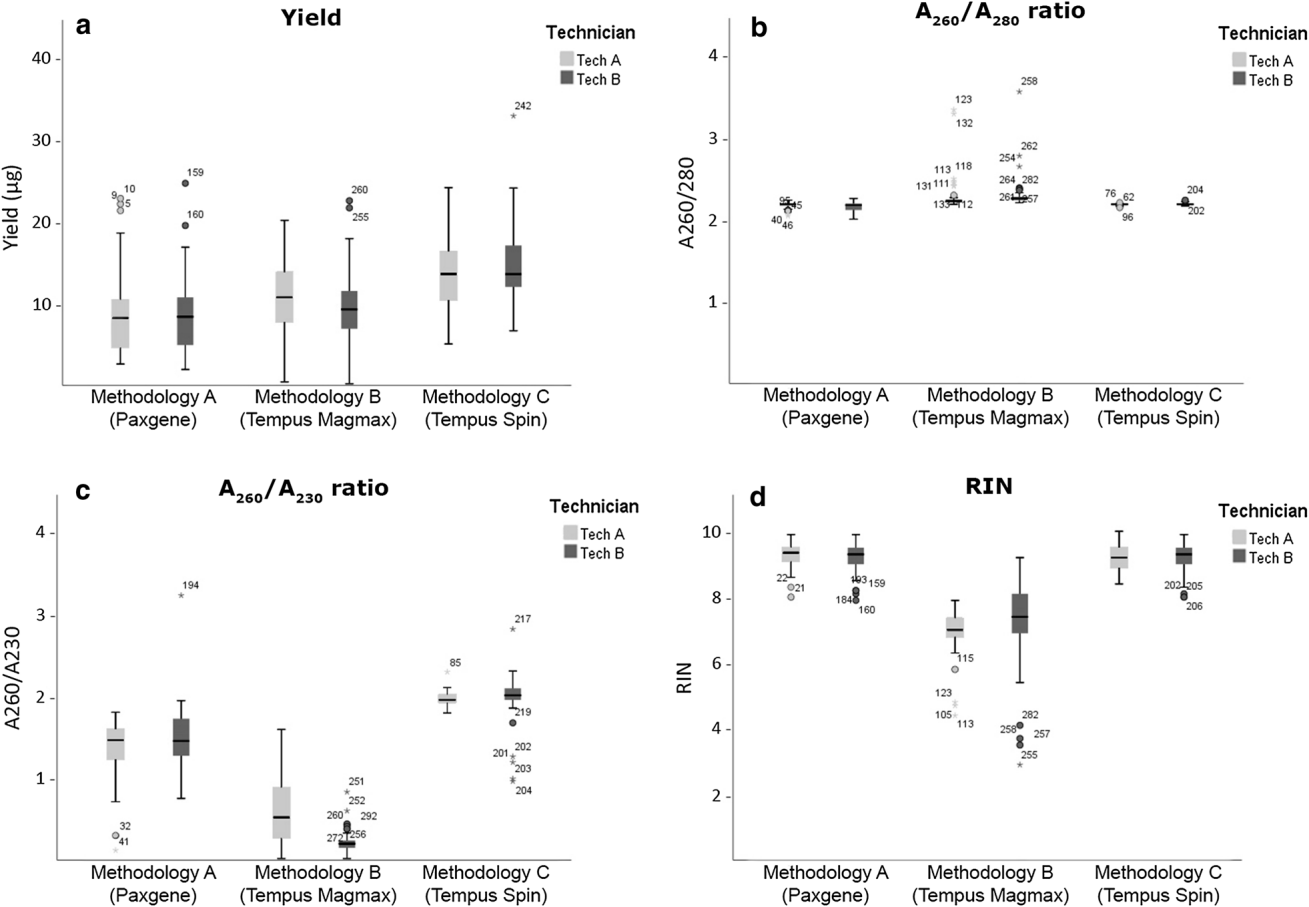
‘RNA yield’ (**a**), ‘A_260_/A_280_’ (**b**), ‘A_260_/A_230_’ (**c**), and ‘RIN integrity’ (**d**) for RNA isolated by the three methodologies with both technicians (Tech A and Tech B). Box-plots of the data are represented. The box with horizontal black line shows the first and third quartiles and the median. The whiskers show 1.5 times the interquartile range. The points show outliers

A comparison of the RNA yield between methodologies and technicians’ were performed. The average yields, are indicated in Additional file 2: Table B and represented in Fig. 2a. The best results were obtained with Methodology C. Significant differences were observed between methodologies and between the two technicians with Methodologies B and C, but not with Methodology A.

#### RNA purity

The A_260_/A_280_ and A_260_/A_230_ ratios were analyzed for the three methodologies (Additional file 2: Table B). Ratios were obtained for all of the samples, except for 5 samples extracted with Methodology B, due to the low RNA concentration of the samples (2.56–16.08 ng/µl). The A_260_/A_280_ and A_260_/A_230_ values obtained are represented in Fig. 2b, c. Methodologies A and C showed the best A_260_/A_280_ ratio. No significant differences were observed between methodologies and technicians.

Regarding the A_260_/A_230_ ratio, large differences were observed between methodologies. The best values were obtained with Methodology C and the worst values were obtained with Methodology B. No significant differences were observed between the technicians for Methodologies A and C, but significant differences were observed for Methodology B (Additional file 2: Table B, Fig. 2b, c).

#### RNA integrity

RIN values were calculated for all of the samples except for 13 samples (6 samples from Methodology A and 7 samples from Methodology B). The 6 samples from Methodology A but for which no RIN values was calculated correspond to samples with high RNA concentrations (118.16–172.16 ng/µl), for which the Bioanalyzer showed an anomalous profile. Those 6 RNA samples were subjected to an extraordinary DNase treatment. The RIN was analyzed again obtaining a good result (Additional file 3). In addition, the 6 original and the 6 aliquots treated with DNase were checked by PCR for the GAPDH gene [18]. PCR amplification was observed in the 6 original samples, whilst the 6 purified samples showed no amplification (data not shown).

The average RIN value and standard deviation are indicated in Additional file 2: Table B and Fig. 2d. The best results were obtained with Methodologies A and C, and no significant differences were observed between methodologies and technicians.

#### Analysis of the reproducibility and robustness of the three methodologies

**Fig. 3:**
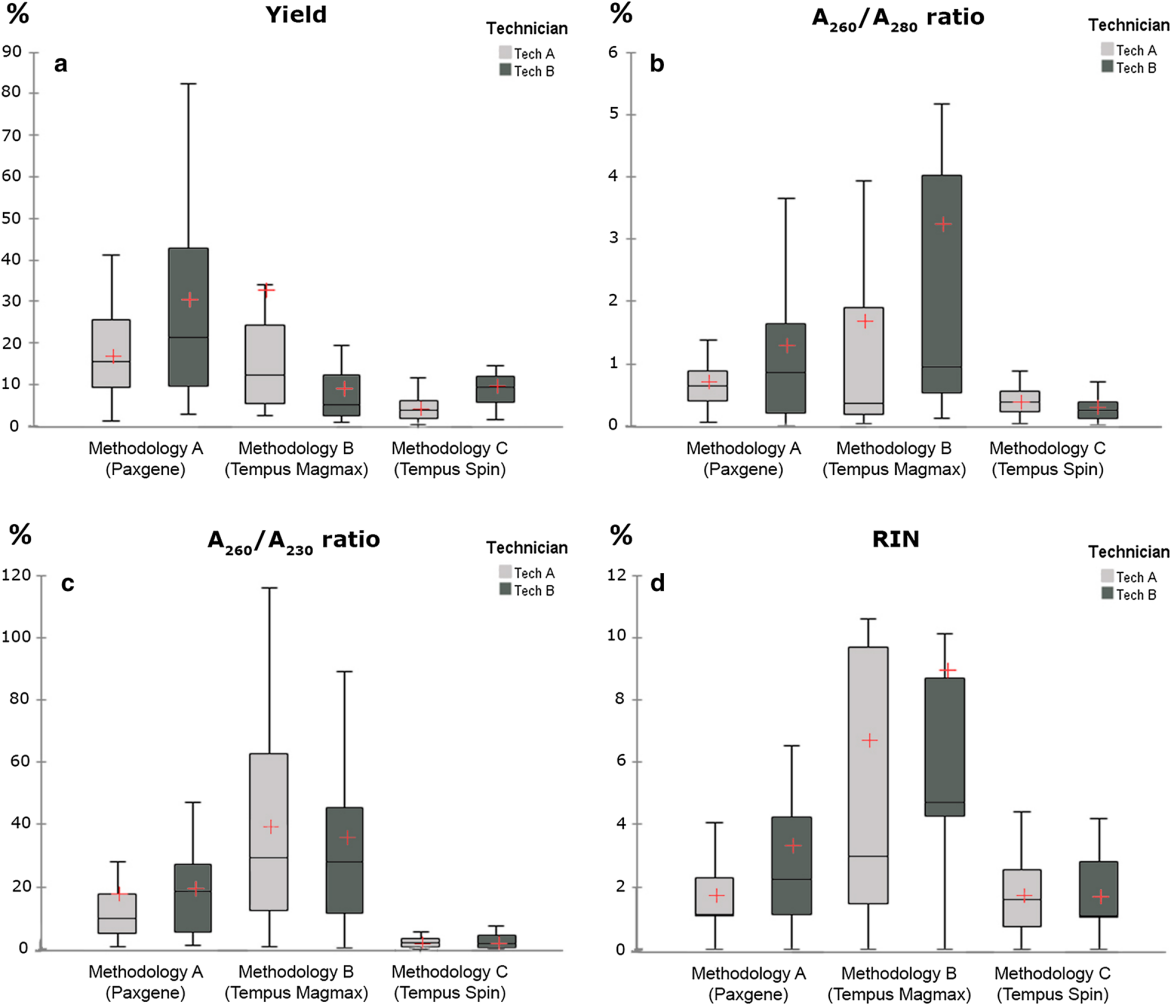
Box-plots for the percentage variation between the replicas for each variable: ‘yield’ (**a**), ‘A_260_/A_280_’ (**b**), ‘A_260_/A_230_’ (**c**), and ‘RIN’ (**d**), with the three methodologies, as tested by both technicians. Box-plots of the data are represented. The box with the horizontal black line shows the first and third quartiles and the median. The whiskers show 1.5 times the interquartile range. The points show outliers. The “+” sign represents the average for each variable

Variability between replicas from the same donor with respect to the three methodologies and both technicians was analysed and the percentage of variation between each pair of samples from each donor that was processed by each technician with each methodology was calculated. The best reproducibility was observed with Methodology C (Additional file 2: Table C and Fig. 3). Only significant differences were observed between technicians for variable “yield” with Methodology C. Technician A obtained better reproducibility results than Technician B with Methodology A. The lowest reproducibility was obtained for both technicians with Methodology B.

#### RNA expression analysis

Forty-two selected samples were used to check the utility of the extracted RNA, considering Ribogreen quantification values. The average Cq and standard deviation values were calculated (Additional file 4). Gene expression was detected in every case. The worst results for mRNA were obtained with methodology A, with significant differences for the 18S rRNA gene and ACTB gene fragments. However, no significant differences were observed for Methodology B and Methodology C.

The best results for miRNA were obtained with Methodology A (especially for mir-30 miRNA), while higher Cq values were observed for Methodology C.

### Discussion

Previous comparative studies have been performed on different methodologies [11]. However, a simultaneous comparison of the proposed methodologies has not been published before. In our study, Methodology C was the most effective with respect to yield, so it provided a yield between 38% and a 56% greater in comparison to Methodologies B and A, respectively. Other studies have shown a higher RNA yield with Tempus tubes [10–12, 19], associated with a smaller volume of blood in the case of PaxGene tubes [5]. However we note that Methodology B also uses Tempus tube and the obtained RNA yield was lower than for Methodology C, so it’s not only important the type of tube, but also the RNA extraction kit. The high degree of heterogeneity that was observed between the donors, with values ranging between 2.88–22.39, 0.43–19.70, and 0.76–24.98 µg for methodologies A, B, and C respectively, is mainly due to the intrinsic characteristics of the samples.

With respect to the purity of the RNA, the best results were obtained with Methodology C. Overall, ‘very good’ and comparable purity values were observed for Methodology A and Methodology C, in accordance with previous research [9, 12, 20]. However, we observed more unstable A_260_/A_230_ ratios with Methodology A and especially for Methodology B than these previous studies. The presence of impurities with Methodology B has been described previously [21]. The differences observed in the A_260_/A_230_ ratio between the two technicians were very marked for Methodology B, possibly due to small differences in the protocol implementation. It has been reported that the observed differences in A_260_/A_230_ ratio with Methodology A, are due to the high salt content of PaxGene kit elution buffer [10].

Methodologies A and C showed extraordinary RNA integrity, not observed in other studies [10–12]. It was not possible to obtain the RIN value for seven samples extracted by using Methodology B due to the low concentration of the samples. The RIN value was also not available for 6 samples that were extracted with Methodology A, because of the anomalous profile. The fact that this occurs with some frequency with paired samples from the same donor suggests some peculiarity of the sample, and Methodology A wasn’t able to eliminate the contaminant. The bioanalyzer fluorogram only shows nucleic acids those are present in the sample [22]. Using an extraordinary DNase treatment and PCR we demonstrated that DNA traces are present, and the kit hasn’t been able to eliminate in during RNA purification procedure, probably because of the high RNA concentration, due to a high cellularity count and a consequent large amount of DNA.

Additionally, Methodology C show greatest robustness between replicas and technicians. The differences observed between technicians, may be attributed to a better understanding and/or execution of a particular protocol. Clearly, there was a huge variability in the purity and integrity measures with Methodology B, possibly due to a non-efficient contaminants removal.

Slightly worse expression results were obtained for mRNAs with Methodology A, although this methodology showed a higher detection capacity for miRNAs. The purification capacity of miRNA for Methodology C should be noted, although the manufacturer doesn’t guarantee the purification of miRNA. Skogholt et al. suggest that differences in expression between methodologies are due to sampling systems, influenced by physical properties of the transcripts [2]. Although 6 transcripts were studied in the present study, we observe correlation between methodologies that use PaxGene and the Tempus tubes with respect to the expression levels of mRNA. However, there is an effect of the extraction protocol on the expression of miRNA, so not only the type of tube is important [11, 19], and the selected methodology influences the mRNA and miRNA expression profiles. It is essential to establish the standardization of sample processing for large cohort studies, such data from mixed methodologies should never be analyzed together [11, 19, 23].

To summarize, superior results were obtained with Methodologies A and C. For Biobank large-cohort and epidemiological studies, Methodology C is the most appropriate methodology. However, the reduced hands-on time that is needed for Methodology A must also be considered.

## Limitations

DNase treatment was included in Methodologies A and B; however the additional treatment with DNase was not performed with Methodology C.

## Supplementary information

**Additional file 1.** (i) Reversotranscription, (ii) mRNA and miRNA real time PCR conditions, (iii) and oligo sequences details.

**Additional file 2:** **Table A.** Summary of the technical characteristics, technical requirements and cost analysis for the three methodologies tested. **Table B.** Average and standard deviation obtained for RNA yield, A260/A280, A260/A230 and RIN obtained by both technicians with the three methodologies. The average values encompassing the values obtained by both technicians are also indicated. **Table C.** Robustness of each methodology analysis. Average percentage and standard deviation for variation between methodologies and technicians for variables yield, A260/A280, A260/A230 and RIN.

**Additional file 3.** Diagram of an RNA electropherogram for six samples purified with Methodology A. Images **a**–**f** correspond to the RNA purified by Methodology A, following the manufacturer’s instructions. The images **a’**–**f’** correspond to the same RNA samples after additional treatment with DNase. **a** and **a’**: Donor 16 Sample 1, **b** and **b’**: Donor 16 Sample 2, **c** and **c’**: Donor 17 sample 1, **d** and **d’**: Donor 23 Sample 1, **e** and **e’**: Donor 23 Sample 2, **f** and **f’**: Donor 23 Sample 3. The anomalous peaks of the electropherograms are shaded in the picture.

**Additional file 4.** Representation of the average Cq value for the RNA obtained by the three methodologies. mRNA genes (18S rRNA, ACTB, and GAPDH) and miRNA (mir-26, mir-30, and mir-16) were tested. Error bars are indicated.

## Data Availability

The datasets used and/or analyzed during this study are available from the corresponding author on request.

## Abbreviations

GAPDH: Human glyceraldehyde-3-phosphate dehydrogenase
18S rRNA: Ribosomic RNA 18S
ACTB: Beta actin

## References

[1] Hebels DG, Georgiadis P, Keun HC, Athersuch TJ, Vineis P, Vermeulen R, Portengen L, Bergdahl IA, Hallmans G, Palli D (2013). Performance in omics analyses of blood samples in long-term storage: opportunities for the exploitation of existing biobanks in environmental health research. Environ Health Perspect.

[2] Skogholt AH, Ryeng E, Erlandsen SE, Skorpen F, Schonberg SA, Saetrom P (2017). Gene expression differences between PAXgene and Tempus blood RNA tubes are highly reproducible between independent samples and biobanks. BMC Res Notes.

[3] Pantel K, Alix-Panabieres C (2010). Circulating tumour cells in cancer patients: challenges and perspectives. Trends Mol Med.

[4] Kim JH, Jin HO, Park JA, Chang YH, Hong YJ, Lee JK (2014). Comparison of three different kits for extraction of high-quality RNA from frozen blood. Springerplus.

[5] Meyer A, Paroni F, Gunther K, Dharmadhikari G, Ahrens W, Kelm S, Maedler K (2016). Evaluation of existing methods for human blood mRNA isolation and analysis for large studies. PLoS ONE.

[6] Chai V, Vassilakos A, Lee Y, Wright JA, Young AH (2005). Optimization of the PAXgene blood RNA extraction system for gene expression analysis of clinical samples. J Clin Lab Anal.

[7] Rainen L, Oelmueller U, Jurgensen S, Wyrich R, Ballas C, Schram J, Herdman C, Bankaitis-Davis D, Nicholls N, Trollinger D, Tryon V (2002). Stabilization of mRNA expression in whole blood samples. Clin Chem.

[8] Tan SC, Yiap BC (2009). DNA, RNA, and protein extraction: the past and the present. J Biomed Biotechnol.

[9] Asare AL, Kolchinsky SA, Gao Z, Wang R, Raddassi K, Bourcier K, Seyfert-Margolis V (2008). Differential gene expression profiles are dependent upon method of peripheral blood collection and RNA isolation. BMC Genomics.

[10] Duale N, Brunborg G, Ronningen KS, Briese T, Aarem J, Aas KK, Magnus P, Stoltenberg C, Susser E, Lipkin WI (2012). Human blood RNA stabilization in samples collected and transported for a large biobank. BMC Res Notes.

[11] Hantzsch M, Tolios A, Beutner F, Nagel D, Thiery J, Teupser D, Holdt LM (2014). Comparison of whole blood RNA preservation tubes and novel generation RNA extraction kits for analysis of mRNA and MiRNA profiles. PLoS ONE.

[12] Menke A, Rex-Haffner M, Klengel T, Binder EB, Mehta D (2012). Peripheral blood gene expression: it all boils down to the RNA collection tubes. BMC Res Notes.

[13] PAXgene Blood miRNA Kit Handbook. Qiagen-Preanalytix ed.; 2015.

[14] MagMAX™ for Stabilized Blood Tubes RNA Isolation Kit Protocol. In: *Part Number 4452007 Rev A* (Technologies A-L ed., vol. Part Number 4452007 Rev. A; 2010.

[15] Tempus™ Blood RNA Tube and Tempus™ Spin RNA Isolation Kit Protocol. D PNR ed. Applied Biosystems; 2008.

[16] Lopez P, Wagner KD, Hofman P, Van Obberghen E (2016). RNA activation of the vascular endothelial growth factor gene (VEGF) promoter by double-stranded rna and hypoxia: role of noncoding VEGF promoter transcripts. Mol Cell Biol.

[17] Boutz PL, Chawla G, Stoilov P, Black DL (2007). MicroRNAs regulate the expression of the alternative splicing factor nPTB during muscle development. Genes Dev.

[18] Kang H, Rho JG, Kim C, Tak H, Lee H, Ji E, Ahn S, Shin AR, Cho HI, Huh YH (2017). The miR-24-3p/p130Cas: a novel axis regulating the migration and invasion of cancer cells. Sci Rep.

[19] Nikula T, Mykkanen J, Simell O, Lahesmaa R (2013). Genome-wide comparison of two RNA-stabilizing reagents for transcriptional profiling of peripheral blood. Transl Res.

[20] Feddersen S, Bastholt L, Pedersen SM (2017). Stabilization of circulating thyroglobulin mRNA transcripts in patients treated for differentiated thyroid carcinoma. Ann Clin Biochem.

[21] Richards J, Unger ER, Rajeevan MS (2019). Simultaneous extraction of mRNA and microRNA from whole blood stabilized in tempus tubes. BMC Res Notes.

[22] Grissom SF, Lobenhofer EK, Tucker CJ (2005). A qualitative assessment of direct-labeled cDNA products prior to microarray analysis. BMC Genomics.

[23] Yip L, Fuhlbrigge R, Atkinson MA, Fathman CG (2017). Impact of blood collection and processing on peripheral blood gene expression profiling in type 1 diabetes. BMC Genomics.

